# Telocytes response to cardiac growth induced by resistance exercise training and endurance exercise training in adult male rats

**DOI:** 10.1186/s12576-023-00868-2

**Published:** 2023-06-10

**Authors:** Siroos Choobineh, Mahboobeh Borjian Fard, Rahman Soori, Zohreh Mazaheri

**Affiliations:** 1 Department of Exercise Physiology, Faculty of Sport Sciences and Health, University of Tehran, Tehran, Iran; 2 Basic Medical Science Research Center, Histogenotech Company, Tehran, Iran

**Keywords:** Telocytes, Endurance training, Resistance training, Cardiac growth

## Abstract

Telocytes are interstitial cells found in different tissues, including cardiac stem cell niches. The purpose of this study was to investigate the response of the telocytes to the cardiac growth that occurs in response to resistance and endurance exercise trainings using rats distributed into control, endurance, and resistance training groups. Results revealed that the ratio of heart weight to body weight, cardiomycyte number, cardiomyocyte area, thickness of the left ventricular wall were significantly higher in the training groups compared to the control group. We observed increment in the cardiomyocytes surface area and thickness of the left ventricular wall in the resistance-training group than endurance-training group. We conclude that both resistance and endurance exercise trainings will lead to an increased number of cardiac telocytes, consequently, promote activity of the cardiac stem cells, and results in physiological cardiac growth, and this response does not seem to depend on the type of exercise.

## Background

The physiological cardiac growth is a response to the exercise training. This growth can be through an increase in cell number (cardiomyocyte hyperplasia) or consequently in cell size (cardiomyocyte hypertrophy) [8]. New cardiomyocytes arise from existing cardiomyocytes or from cardiac stem cells [27]. Usually, cardiac stem cells exist in stem cell niches, special microenvironments located in depth of tissues, which nurture stem cells and control their behavior [23]. The fate of a cardiac stem cell is governed by the niche components, including cardiac telocytes.

Telocytes recently discovered in intestinal cells and characterized by small cell body and numerous lengthy cytoplasmic prolongations, telopodes, which differs from other classic stromal cells [41]. Additionally, there are certain dissimilarities between the expression patterns of telocytes and other stromal cells. Telocytes also display different immunophenotypes [7]. These cells can be found in different tissues and organs (e.g., heart) [24].

Cardiac telocytes are widely found in the epicardium, myocardium, endocardium, heart valves, and notably in the cardiac stem cell niches, adjacent to the stem cells and progenitor cells [41]. Their presence in this site further elucidates their role in homeostasis, activation, and differentiation of cardiac stem cells [7, 24]. Zhao and colleagues reported that the number of cardiac telocytes decreases after myocardial infarction. They also found that transplantation of cardiac telocytes reduces infarct size and improves myocardial function of infarcted cardiac [49]. In addition, considering their role in the stem cell activation, increasing the number of cardiac telocytes could be a novel strategy to improve the regeneration of the aging cardiovascular system [7].

Given the function of cardiac telocytes in niches and their role in cardiac rehabilitation and regeneration, the response of cardiac telocytes to exercise training that regulates the physiological cardiac growth, is interesting. In a recent study, Xiao and colleagues used endurance swimming training to induce cardiac growth. They concluded that the endurance training promotes the formation of new telocytes, this induces an increase in the physiological cardiac growth as a response to the exercise training [46]. However, the rate of cardiac growth and extension of physiological and morphological changes depend on the type of exercise [44].

Studies revealed that both resistance exercise training [14, 35], Melo et al. [34]) and the endurance exercise training [10, 30, 48] lead to physiological growth of the heart, but their mechanisms are different. Due to the volume overload, endurance exercise training causes cardiac eccentric hypertrophy, that is, increased left ventricular volume and proportional changes in left ventricular wall thickness. In contrast, owing to the pressure overload, resistance exercise training results in concentric hypertrophy and thickening of the left ventricular wall without alteration in the left ventricular cavity [37].

Despite the differences in the mechanisms of cardiac growth caused by the resistance and endurance training, impact of these types of training methods, especially resistance training, on cardiac telocytes it is not known. Thus, the purpose of this study is to investigate the response of telocytes to cardiac growth induced by two types of exercises, resistance, and endurance training.

## Method

In the present experimental study, 24 Wistar male rats (8 weeks old and body weight, of 217 ± 14 g) were randomly divided into three equal groups: (1) control (*n* = 8), (2) resistance training (*n* = 8), and 3) endurance training (*n* = 8). The rats were housed in the animal care facilities under the same standard laboratory conditions of light (12 h light, 12 h dark cycle), temperature (22 ± 3 °C), and humidity (50% ± 10%) with free access to standard laboratory food and water. The Wistar rats were kept in this condition to the experiments to be accustomed to the environment. (Ethical approval for this study was obtained from the Research Ethics Committee of Sport Sciences Research Institute of Iran. ID: IR.SSRI.REC.1397.276.)

### Exercise trainings

Animals were introduced to the exercise protocols for 1 week. Those of the resistance-training group were trained to climb a 1-m vertical ladder (85° incline) with an extra load attached to their tails. The weights were progressively increased over the course of 8 weeks: from 30 to 200% of their body weight. The rats were placed at the bottom of the ladder and stimulated to climb with weights secured to their tails. They had five training sessions per week, each session consisting of three sets of repetitions of the mentioned exercise. A 1 and 3-min resting period was included between each repetition and set, respectively. Each training session started and ended with a warming up and cooling down set, consisted of five sets of repetitions of the mentioned exercise without any weights [26].

Prior to the endurance training, maximal oxygen uptake (VO2max) was measured by indirect yet accurate calorimetry [19]. The rats were then trained to a percentage of VO2max as specified by Kemi and colleagues. After 5–10 minutes of warming up with 50% to 60% of VO2max, the endurance-training group practiced continuously for 50 min with 65–70% of VO2max. A training session terminated with a cooling down period, similar to the warming up period [21]. Concurrently the control group received no training.

### Assessment of cardiac growth

At last, 48 h after the last training session, rats were anesthetized with a combination of ketamine (50 mg/kg) and xylazine (5 mg/kg). Heart weight-to-body weight ratio was used as a parameter of reflecting cardiac growth. Furthermore, left ventricular wall thickness and cardiomyocyte size and number were calculated by hematoxylin–eosin staining and α-actin staining. Hematoxylin–eosin staining is commonly used histological technique that allows visualization of tissue structures and counting cells [2, 4, 40]; also, immunofluorescent staining of α-actinin can provide an indirect estimate of cardiomyocyte number [17]. For this purpose, body weights of rats were measured after induction of anesthesia. The animal's hearts were excised and weighed. Parts of the ventricular tissues were snap-frozen in liquid nitrogen and stored at − 80 °C for cellular and molecular testing; other parts of the ventricular tissues were immersed in neutralized formalin for immunohistochemical analysis.

The left ventricular wall thickness, cardiomyocyte size and number were assessed under a light microscope. With this aim the ventricular tissue sections at a thickness of 5 µm were prepared by using a microtome and stained with hematoxylin–eosin. Five randomly different microscopic fields of stained heart tissue sections were captured and analyzed by ImageJ 1.48v version software.

Immunofluorescent staining of α-actinin was performed to evaluate changes in the number of cardiomyocytes. Ventricular tissues were fixed and dehydrated with ethanol, and then sliced into 5-μm sections using a microtome. The samples were washed with PBS for 5 min and incubated with 0.3% Triton for 30 min to permeabilize the cytomembrane. To prevent non-specific binding of antibodies, the samples were exposed to 10% goat serum for 30 min. Rabbit antibody to α-actinin (Biorbyt, UK) was added to the sample and incubated overnight in a humid environment. The following day, the sample was washed four times with PBS before adding secondary antibodies at a dilution of 1:150 and incubating at 37 °C for 1 h and 30 min in the dark. Propidium iodide was added to confirm the markers, and the sample was observed using an Olympus Fluorescent microscope with a 400 lens.

### Evaluation of BNP mRNA and ANP mRNA

Pathological cardiac hypertrophy is a response to stimuli such as high blood pressure, valvular disease, myocardial infarction, and gene mutation [33]. It is often associated with the upregulation of fetal genes, including brain natriuretic peptide (BNP) and atrial natriuretic peptide (ANP). In contrast, physiological cardiac hypertrophy induced by exercise training is associated with normal heart structure and function and does not increase the activity of fetal genes ANP and BNP [39]. To ensure that the cardiac growth caused by 8 weeks of exercise training was physiological rather than pathological, we determined the expression levels of ANP and BNP using quantitative real-time polymerase chain reaction (qRT-PCR), which are two physiological markers of cardiac growth [33]. The primers were designed based on the sequences in the GenBank^®^ sequence database. The primers are listed in Table 1. RNA was then extracted and converted to cDNA. The cDNA was amplified by PCR and the expression of the two mentioned genes was evaluated. Glyceraldehyde-3-phosphate dehydrogenase (GAPDH), a housekeeping gene, was used as a reference gene for the data normalization. The temperature program used in real-time PCR includes 95° C for 10 min in the heating stage of the sample placement plate and thermal cycles including 95 °C for 15 s, 60 °C for 1 min (repeat 40 cycles). The melting curve was used to confirm the specificity of primers. The relative expression levels of ANP and BNP were calculated using the ${2}^{-\mathrm{\Delta \Delta }C}T$ method and normalized using the GAPDH as the reference [31].

**Table 1:** Primer sequences used in qRT-PCR

	Forward primer sequence [5′-3′]	Reverse primer sequence [5′-3′]
Gapdh	CAT ACT CAG CAC CAG CAT CAC C	AAG TTC AAC GGC ACA GTC AAG G
ANP	CCTGGACTGGGGAAGTCAACC	CTGGGCTCCAATCCTGTCAATCC
BNP	CTGGGCTCCAATCCTGTCAATCC	GTGGGAAGTTTGTGCTGGAAGA

### Identification of cardiac telocytes

There are two common ways to identify telocytes, transmission electron microscopy (TEM) and immunohistochemistry, with about 80% of agreement [13]. TEM is more common but costly and time consuming than immunohistochemistry (Kraus et al. [25]). Hence, like several other researchers [16, 29, 46, 52], we used Immunohistochemistry in our work.

We used dual immunofluorescence staining for CD34/PDGFRα and CD34/PDGFRβ to analyze the changes in telocytes number induced by the exercise training. Briefly, after the primary fixation and dehydration by ethanol, ventricular tissues were sectioned using a microcode at a thickness of 5 μm. The samples were then washed in PBS in for 5 min. Then, to penetrate the cytomembrane, samples were incubated with 0.3% Triton for 30 min; and exposed for 30 min to 10% goat serum to block non-specific binding of the antibodies. The rabbit polyclonal antibody to PDGFR-α (Biorbyt, UK), rabbit polyclonal antibody to PDGFR-β (Biorbyt, UK) and rabbit polyclonal antibody to CD34 (Biorbyt, UK) were added to the sample and incubated in a refrigerator at 2–8 °C for overnight in a humid environment to prevent the tissue from drying out. The next day, the container containing the tissue was removed from the refrigerator and then washed 4 times with PBS for 5 min each time. Then, secondary antibodies were added with dilution 1–150 and then incubated at 37 °C for 1 h and 30 min in the dark. The sample was then transferred from the incubator to a dark room, and after washing 4 times, propidium iodide (PI; Sigma, UK) was transferred to them, it was immediately removed and poured on the PBS sample. In the last step, the sample was observed by the Olympus Fluorescent microscope with a 400 lens to confirm the markers [46].

### Statistical analysis

We used SPSS statistical software (version 20) for data analysis. Moreover, quantitative data were presented as mean ± SEM and a p-value less than 0.05 was considered as statistically significant. In the first step, the normality of the distribution of the variables was evaluated using the Shapiro–Wilk test. Levene’s test for equality of variances used for the homogeneity of variance assumption. Then, a one-way ANOVA were used to compare the means of the three groups. When significant differences were found, a Tukey’s post hoc test was used to determine the exact location of the differences.

## Results

**Fig. 1:**
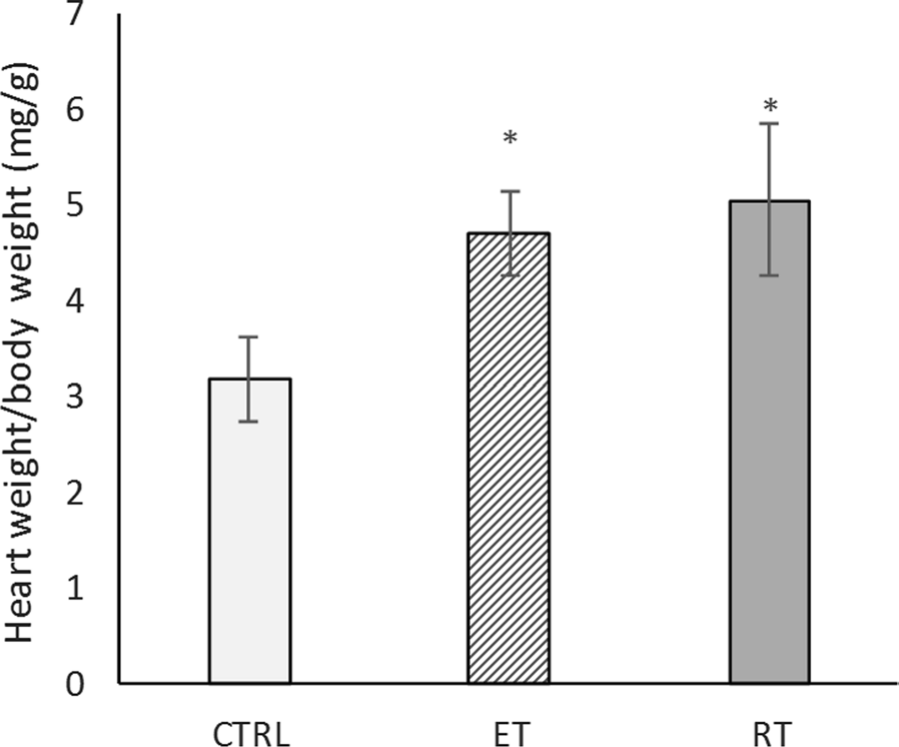
Changes in heart-to-body weight ratio in different groups. (*: significantly different from the control group. *CTRL* control group, *ET* endurance training group, *RT* resistance training group)

The results, as shown in Fig. 1, revealed that the heart weight-to-body weight ratio in both the endurance training and the resistance training groups was significantly higher than the control group (*P* = 0.000 and *P* = 0.000, respectively). Thus, with respect to heart weight-to-body weight ratio, cardiac hypertrophy was observed following the exercise training. Note that the mean weight of the control group was insignificantly higher than the training groups (*P* = 0.1). Table 2 presents heart weights, body weights, and heart-to-body weight ratios.

**Table 2:** Heart weight, body weight and heart weight to body weight ratio

Groups	Body weight(g)	Heart weight(g)	Heart weight/body weight (mg/g)
Control	320 ± 23	1.01 ± 0.11	3.17 ± 0.44
Endurance	285 ± 31	1.33 ± 0.11	4.71 ± 0.43
Resistance	307 ± 35	1.53 ± 0.81	5.05 ± 0.79

**Fig. 2:**
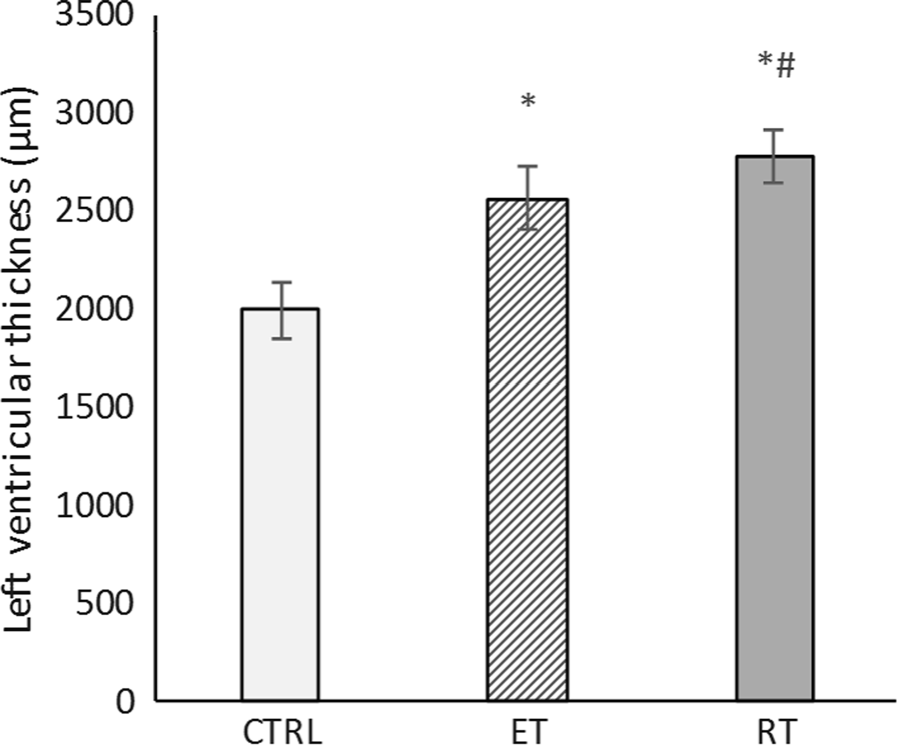
Changes in left ventricular thickness in the studied groups. (* and #: significantly different from the control and the endurance training groups, respectively. *CTRL* Control group, *ET* Endurance training group, *RT* Resistance training group)

**Fig. 3:**
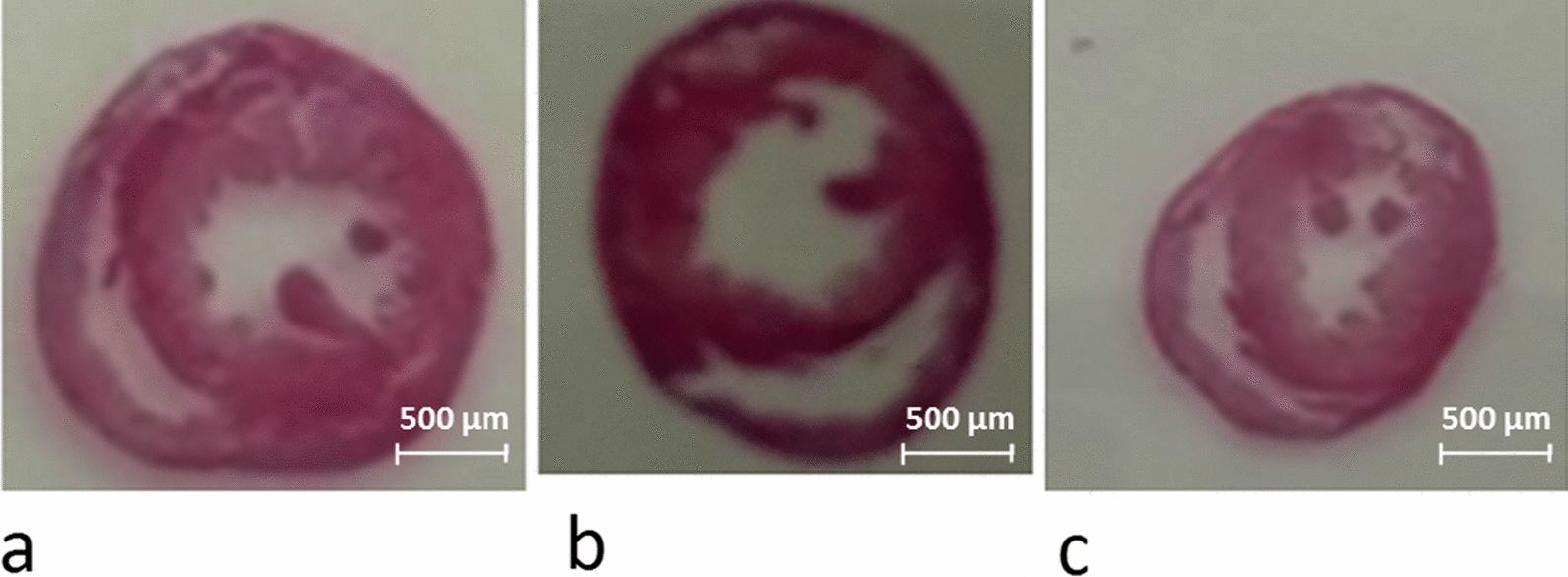
The hematoxylin eosin staining of heart tissue indicated that after 8 weeks of exercise training, the thickness of the left ventricle increased in the rats of the resistance (**a**) and endurance (**b**) groups compared to the control group (**c**)

Measuring the left ventricular wall thickness reveals that both endurance and resistance training results in cardiac hypertrophy (Fig. 2). Hematoxylin and eosin staining showed that the mean left ventricular wall thickness was significantly increased in 8 weeks trained rats compared to the controls (*P* ≥ 0001). The thickness of the left ventricle in the resistance training group was significantly higher than that of the endurance training group (*P* = 0.04). That is, hypertrophy was induced by both endurance and resistance training, which was determined by evaluating left ventricular wall thickness in the groups (Figs. 2 and 3).

**Fig. 4:**
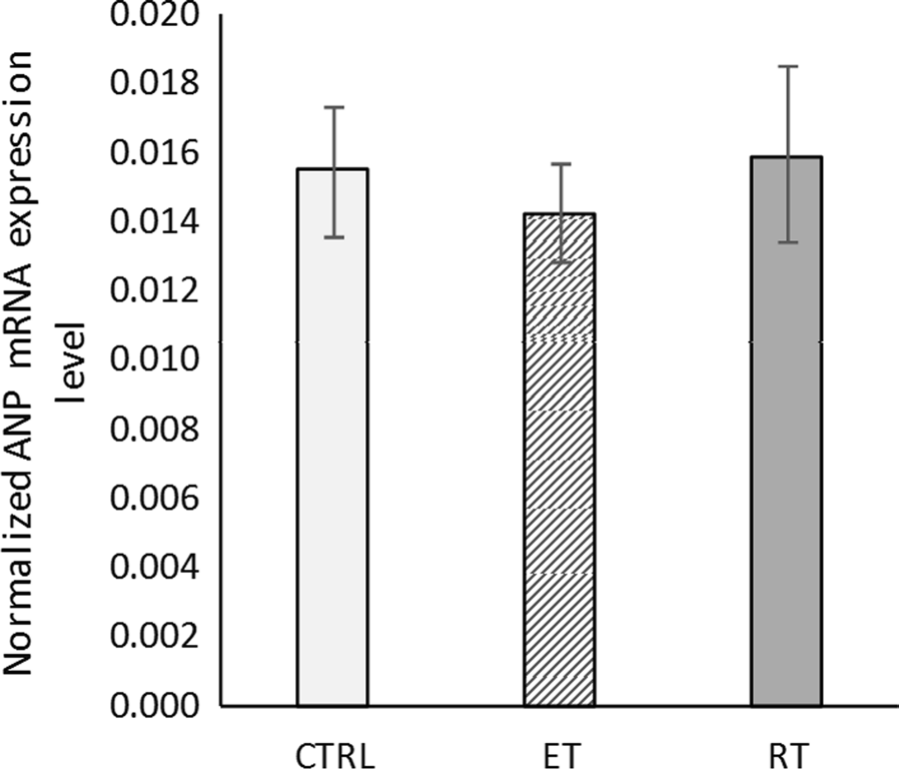
The ANP mRNA levels in different groups. (*CTRL* control group, *ET* endurance training group, *RT* resistance training group)

**Fig. 5:**
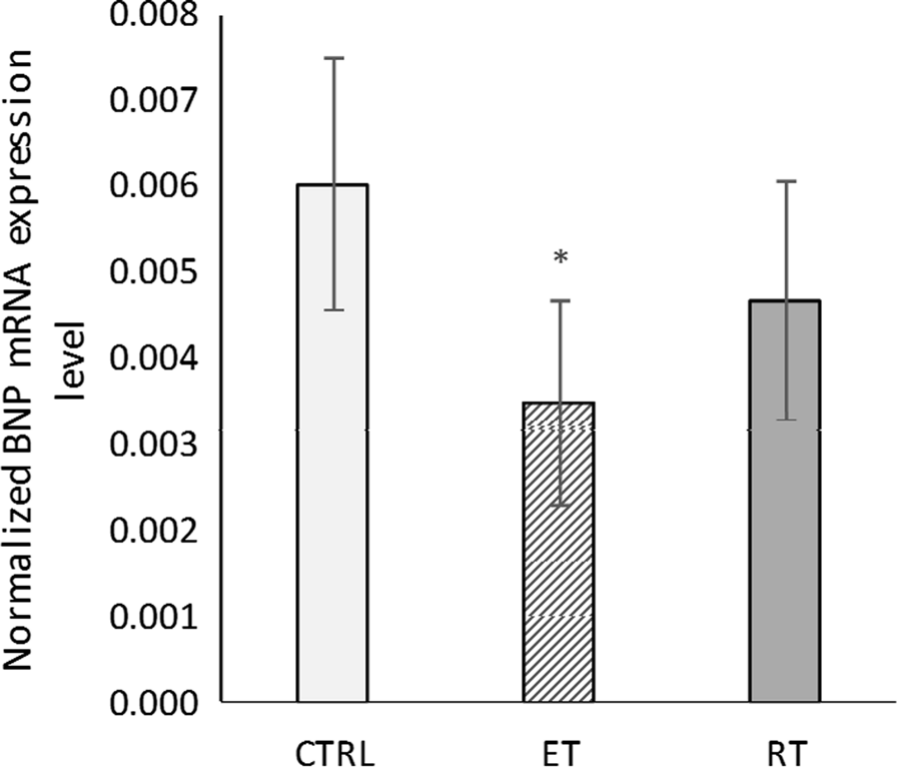
The BNP mRNA levels in different groups. (*: significantly different from the control group. *CTRL* control group, *ET* endurance training group, *RT* resistance training group)

The levels of ANP mRNA did not change significantly after 8 weeks of exercise training (*P* = 0.26) (Fig. 4). Although a significant decrease in BNP mRNA levels were observed in the endurance group compared to the control group (*P* = 0.005), decrease observed in the resistance group was insignificant (*P* = 0.19) (Fig. 5). The ANP mRNA and BNP mRNA levels did not increase significantly, confirming that the hypertrophy was physiological rather than pathological [33]. Therefore, we conclude that 8 weeks of endurance and resistance exercise training causes physiological cardiac growth.

**Fig. 6:**
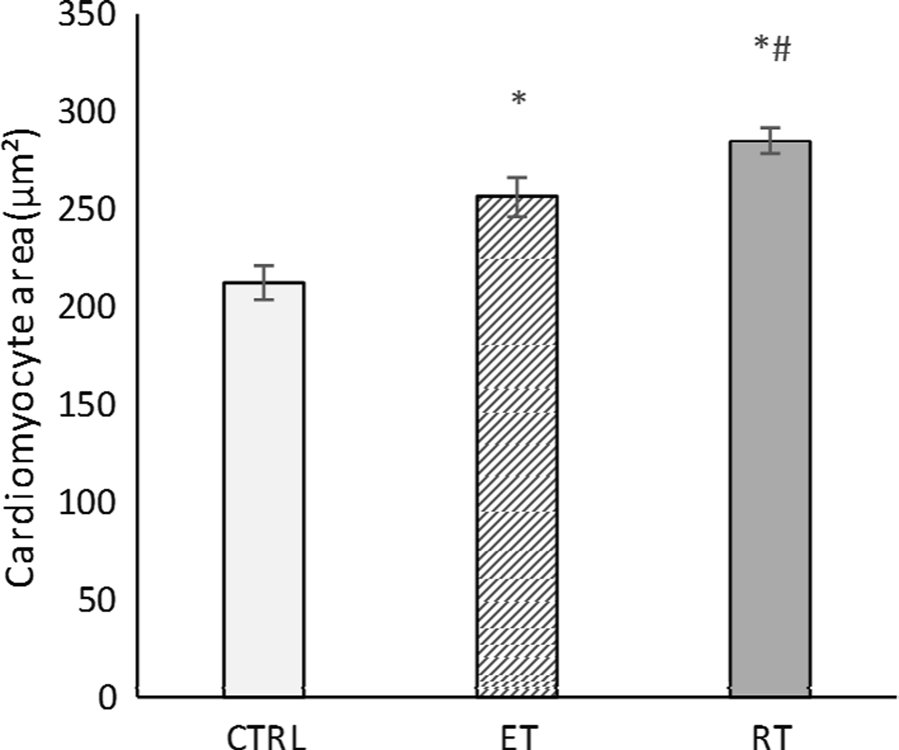
The mean area of cardiomyocytes for the different groups. (* and #: significantly different from the control and the endurance-training groups, respectively. *CTRL* control group. *ET* endurance training group, *RT* resistance training group)

**Fig. 7:**
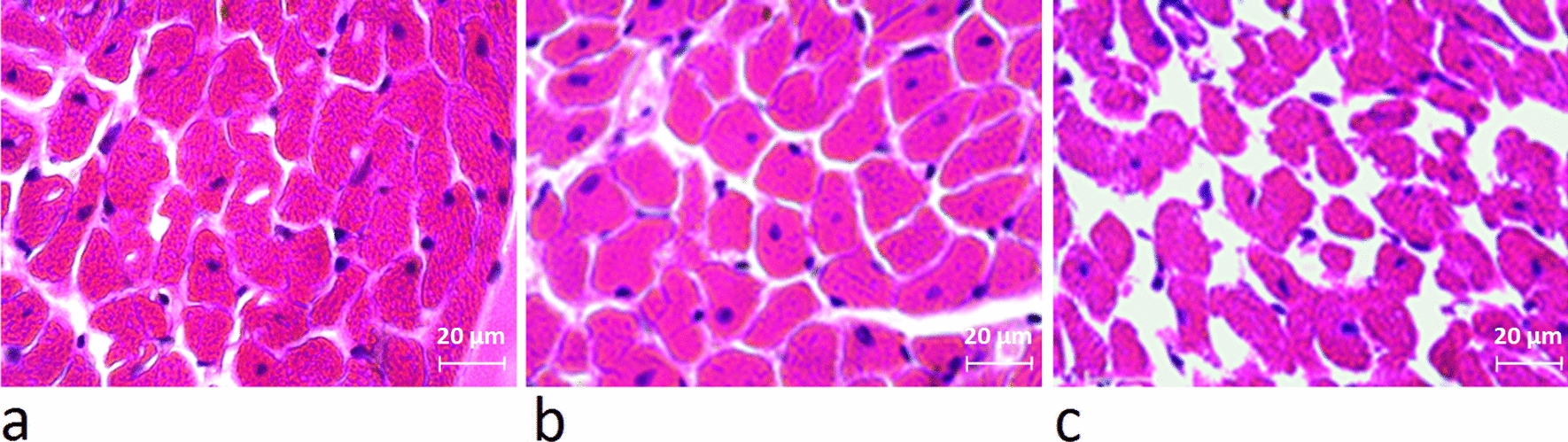
The hematoxylin eosin staining of heart tissue indicated that after 8 weeks of exercise training, area of cardiomyocytes in the resistance (**a**) and endurance (**b**) groups were increased, in compared to the control group (**c**)

Since an increase in size or number of cardiomyocytes, strongly indicates physiological cardiac growth, hence hematoxylin eosin staining was performed to assess the area of cardiomyocytes in heart tissue sections. As shown in Fig. 6, cardiomyocytes area was significantly increased in 8 weeks trained rats compared to the controls, also the increase in cardiomyocytes area in the resistance-training group was significantly higher than that in the endurance-training group (*P* = 0.000) (Figs. 6 and 7).

**Fig. 8:**
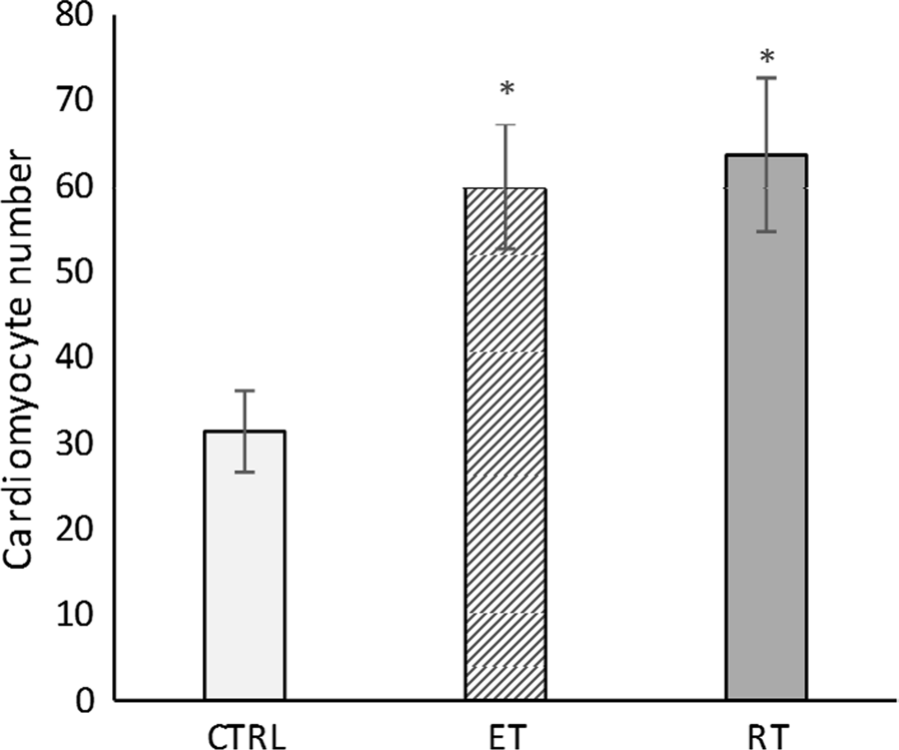
The mean number of cardiomyocytes after 8 weeks of training. (*: significantly different from the control group. *CTRL* control group, *ET* endurance training group, *RT* resistance training group)

**Fig. 9:**
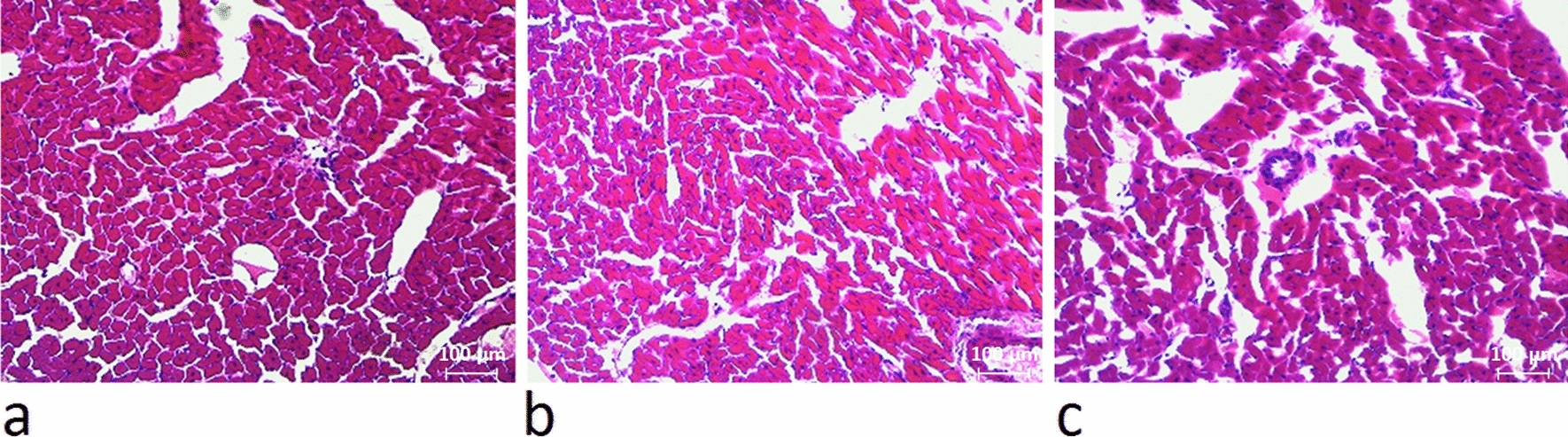
The hematoxylin eosin staining of heart tissue indicated that after 8 weeks of exercise training, the number of cardiomyocytes in the resistance (**a**) and endurance (**b**) groups were increased, in compared to the control group (**c**)

Moreover, hematoxylin eosin and α-actin staining revealed that training could activate production of new cardiomyocytes after 8 weeks, as shown in Figs. 8 and 9, the number of cardiomyocytes in both training groups were significantly higher than that in the control group (*P* = 0.000 and *P* = 0.000, respectively).

**Fig. 10:**
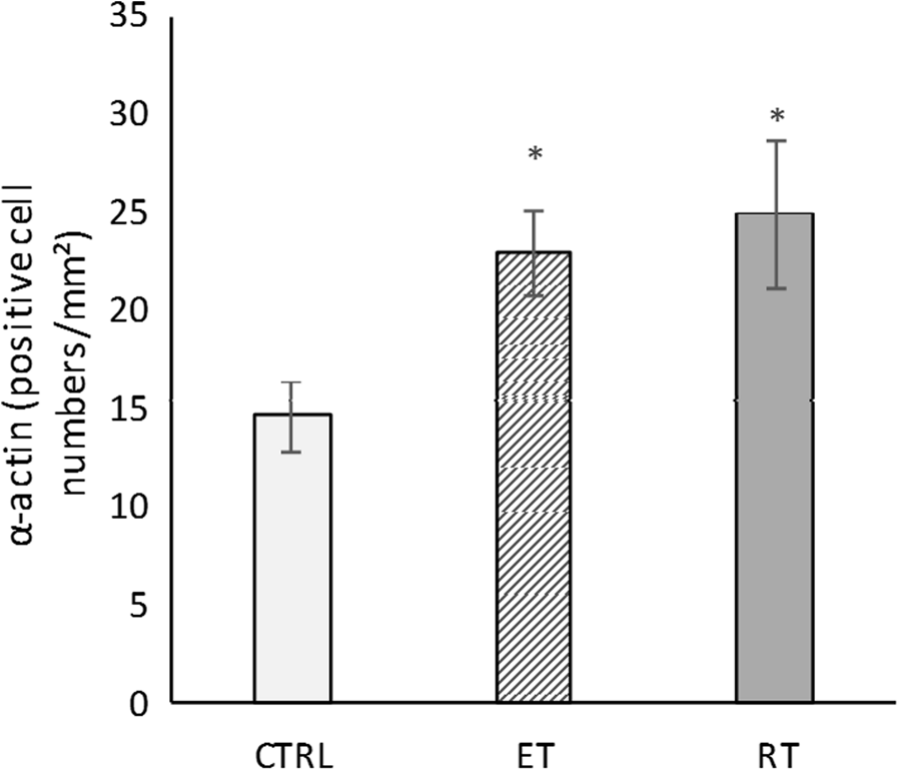
α-actin levels in studied groups. (*: significantly different from the control group. *CTRL* control group, *ET* endurance training group, *RT* resistance training group)

**Fig. 11:**
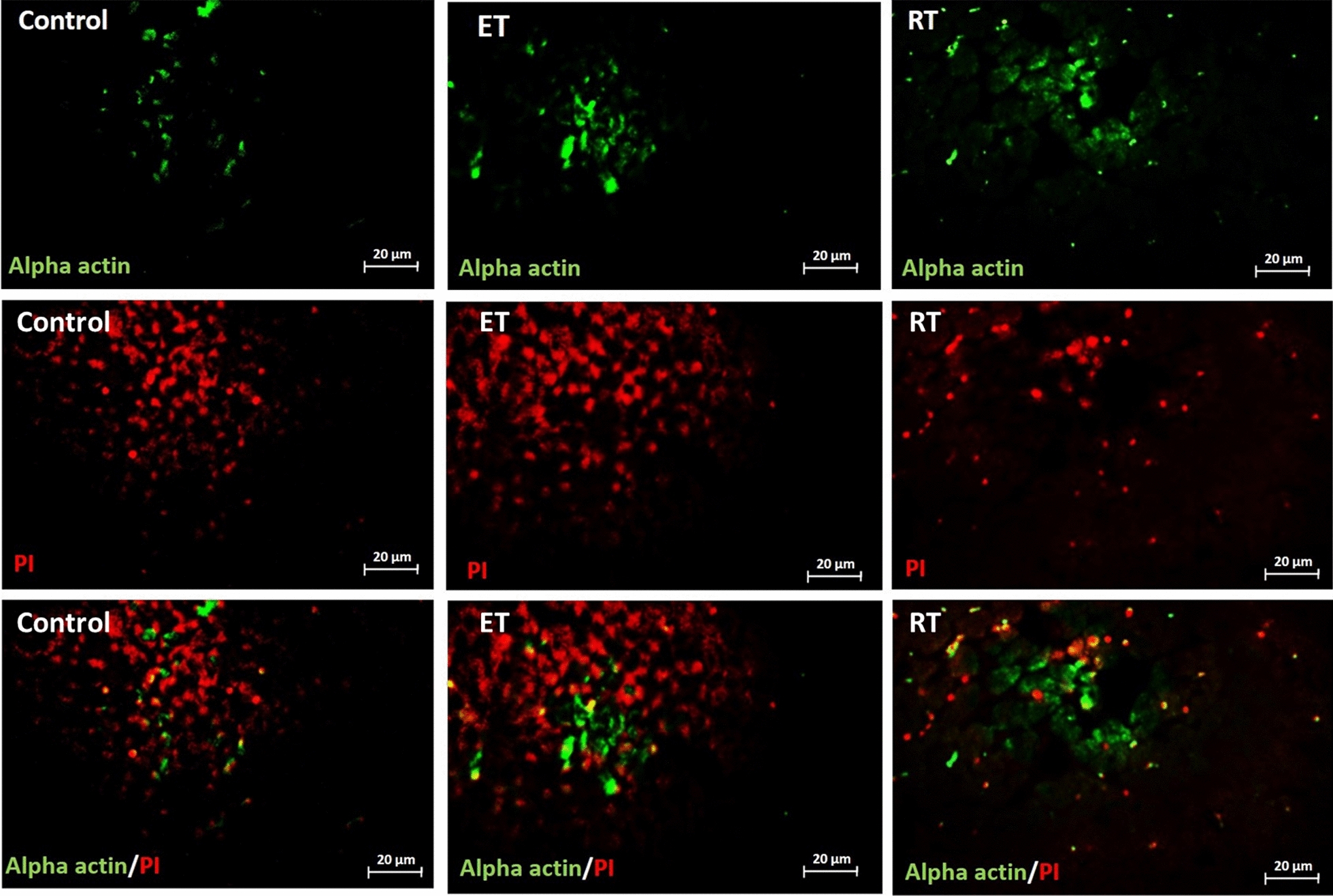
Changes in telocytes number in response to exercise. Double-immunofluorescence labeling for α-actin used to determine telocytes: these cells are increased in response to 8 weeks of endurance training (ET) and resistance training (RT)

Alpha-actin levels were significantly higher in both endurance and resistance training groups compared to the control group (respectively, *P* = 0.02 and *P* = 0.009) (Figs. 10 and 11).

**Fig. 12:**
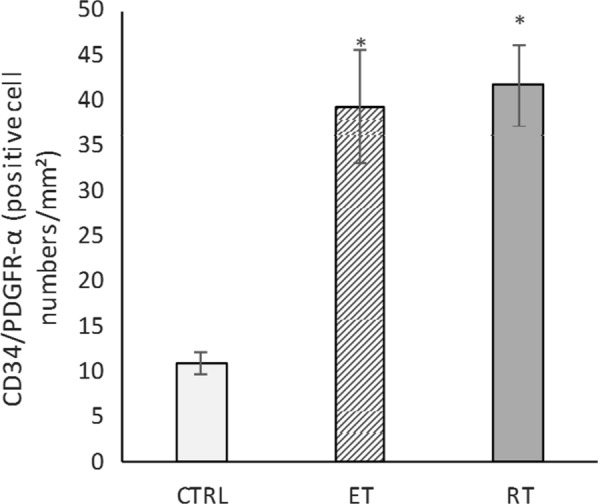
CD34/PDGFR-α levels in studied groups. (*: significantly different from the control group. *CTRL* control group, *ET* endurance training group, *RT*: resistance training group)

**Fig. 13:**
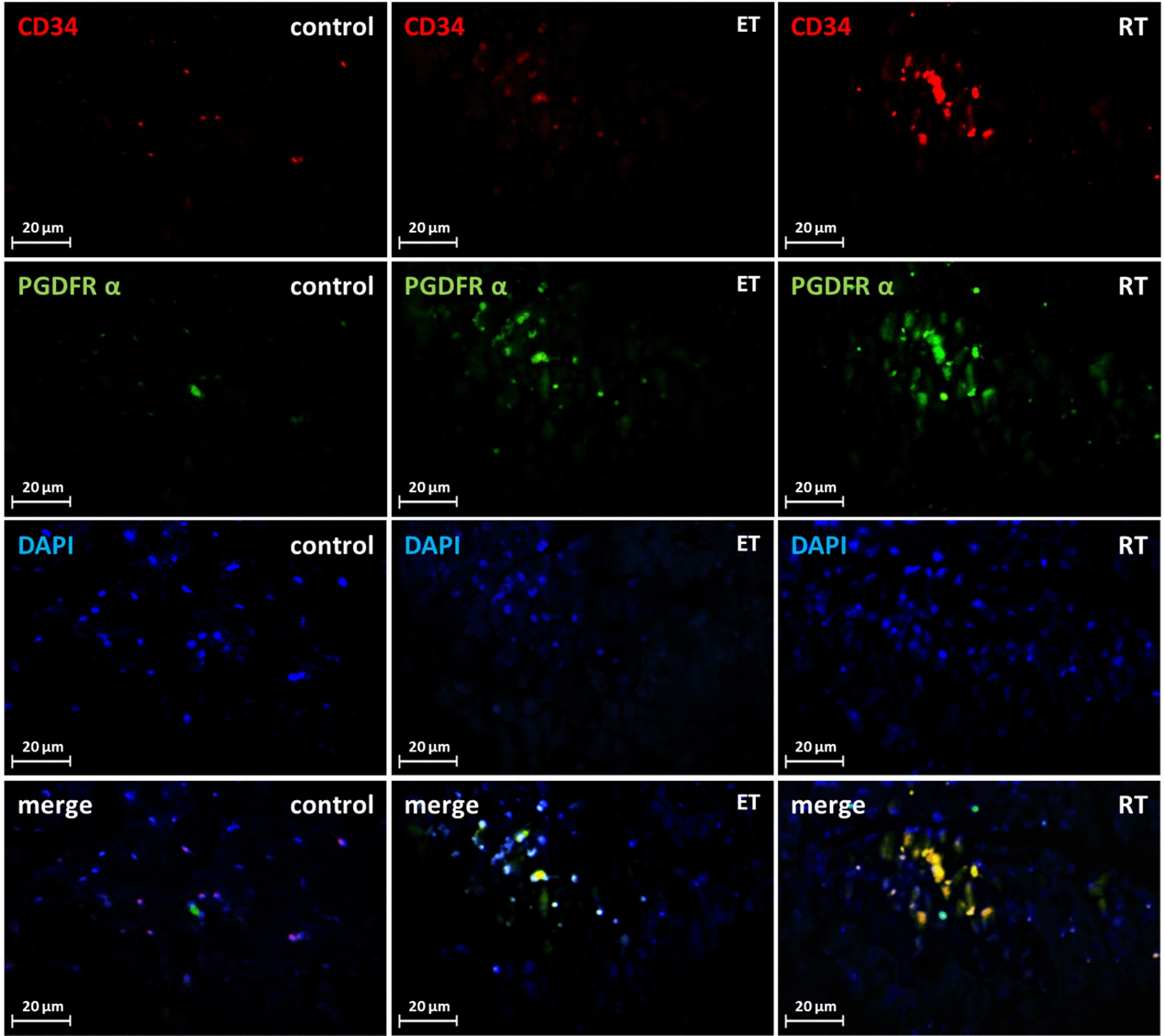
Changes in telocytes number in response to exercise. Double-immunofluorescence labeling for CD34/PDGFR-α used to determine telocytes: these cells are increased in response to 8 weeks of endurance training (ET) and resistance training (RT)

**Fig. 14:**
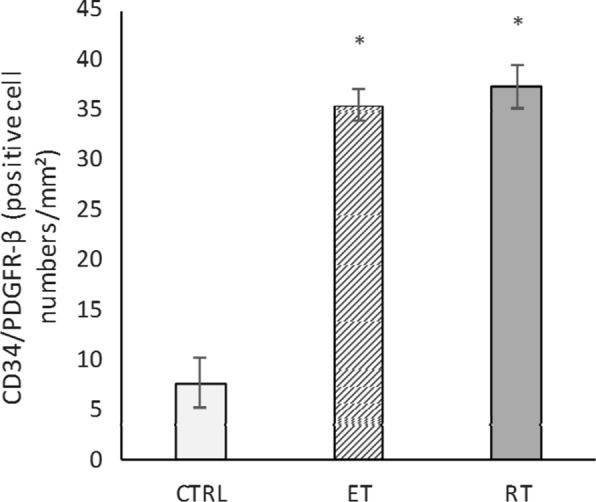
CD34/PDGFR-β levels in studied groups. (*: significantly different from the control group. *CTRL* control group, *ET* endurance training group, *RT* resistance training group)

**Fig. 15:**
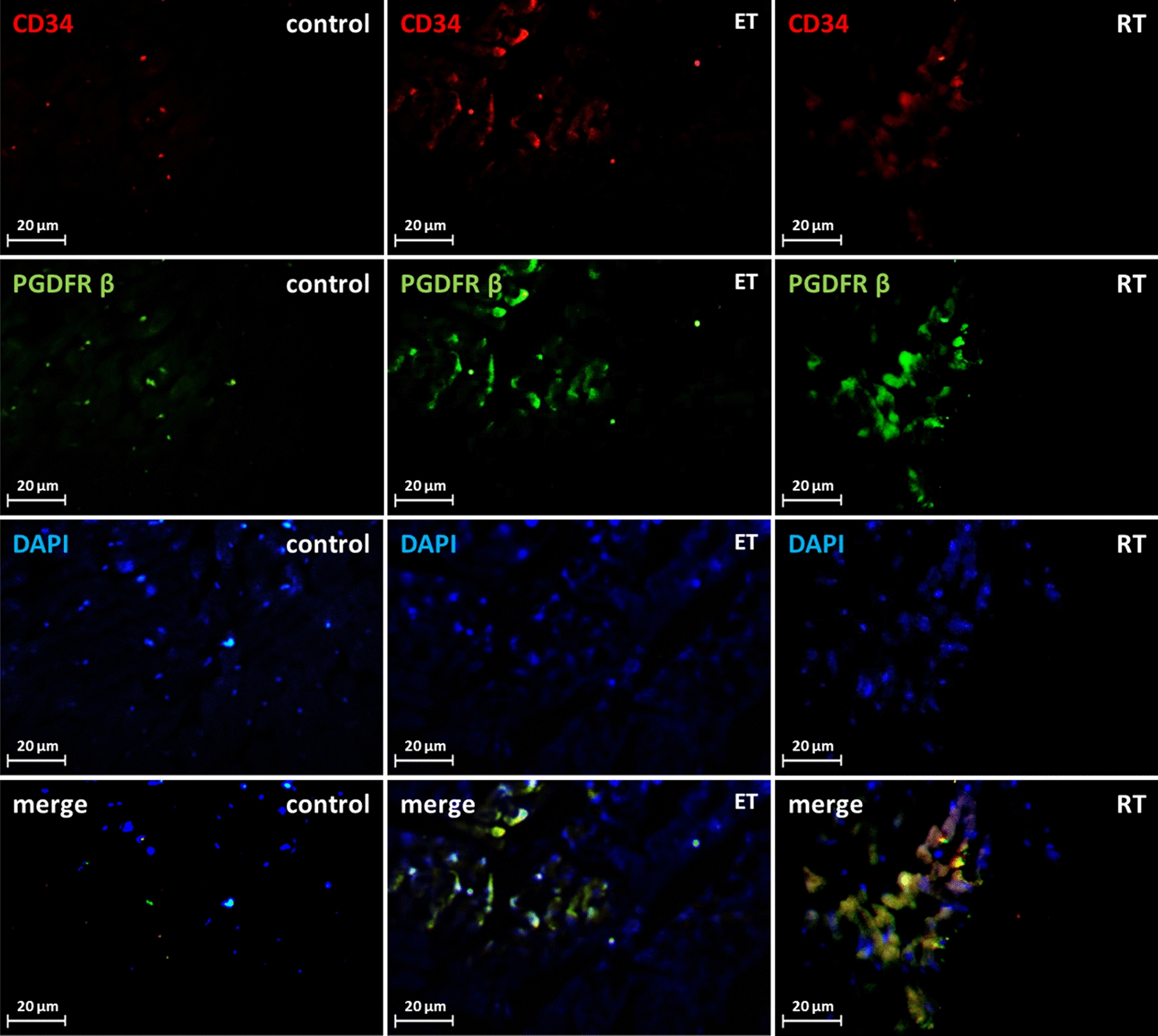
Changes in telocytes number in response to exercise. Double-immunofluorescence labeling for CD34/PDGFR-β used to determine telocytes: these cells are increased in response to 8 weeks of endurance training (ET) and resistance training (RT)

Dual CD34/PDGFRα, CD34/PDGFRβ double staining was used to determine the change in the number of telocytes in the present study. The CD34/PDGFRα levels in the endurance (*P* = 0.000) and resistance (*P* = 0.000) training group were significantly higher than the control group (Fig. 12). Compared to the control group, CD34/PDGFRβ levels were significantly higher in both exercise groups (*P* = 0.000) (Fig. 13). Collectively, these results showed a significant increase in the number of telocytes in the heart of trained rats (Fig. 14 and 15). On the other hand, there was no significant difference between the values ​​obtained from the resistance and endurance groups.

## Discussion

The experience on the Wistar male rats in the present work indicates the physiological growth of the heart followed by the resistance and endurance training. Our results showed an increase in the ratio of heart weight-to-body weight in the training groups compared to the controlled group. Due to the endurance and resistance training, left ventricular hypertrophy (evaluated by their thickness in each group) occurred.

The ANP mRNA and BNP mRNA levels did not increase after 8 weeks of resistance and endurance training. This is while for the endurance training group, the BNP mRNA level decreased significantly. The cardiac hypertrophy seems to be physiological, since this kind of hypertrophy will not upregulate embryonic genes such as ANP and BNP [33].

The main features of physiological cardiac growth caused by exercise increase in the size of cardiomyocytes and the production of new cardiomyocytes [27]. Using hematoxylin and eosin staining and alpha-actin assays, we detected formation of new cardiomyocytes and a significant increase in cardiomyocytes sizes in both exercise groups. Therefore, 8 weeks of resistance and endurance training caused the physiological growth of the heart.

Our findings in the endurance training group are consistent with the results of Liu et al. [30], Xiao et al. [46], Shi et al. [38], Bostrom et al. and [10]. Liu et al. investigated the effect of 3 weeks of swimming and voluntary running on treadmill on exercise-induced cardiac growth in C57 mice. They showed that both exercise models were associated with a decrease in expression of ANP and BNP mRNAs and increase in the HW/BW as well as an increase in the ratio of heart weight to the tibia bone length [30]. Shi et al. reported that 21 days of swimming and voluntary running on the wheel would increase the HW/BW ratio, area, and number of cardiomyocytes in both training groups [38]. Xiao et al. [46] examined the HW/BW ratio, measured size and number of cardiomyocytes after 4 weeks of swimming training in c57 mice. An increase in the heart growth factors in the training group was observed [46]. Bostrom et al. confirmed that the swimming would increase the number of cardiomyocytes and their area by 45% as C/EBPβ decreases and would lead to cardiac growth [10].

Physiological growth of heart induced by the resistance training is consistent with the previous studies [5, 6, 15, 18, 34]. Junqueira et al. [20] did not observe a significant change in the HW/BW ratio and the area of ​​cardiomyocytes of rats trained for 30 days. These findings are not in line with the results of the present study. This discrepancy might be due to the shorter training period. We have shown that the rate of increase in the left ventricular thickness and area of ​​cardiomyocytes in the resistance training group is significantly higher than that of endurance training group. After 8 weeks training, De souza et al. also reached similar results: in comparison to the control group, left ventricular thickness and area of the ​​cardiomyocytes in the endurance and resistance training groups were increased by 6% and 17%, and by 1% and 27%, respectively. Thus, resistance training is more effective in triggering cardiac hypertrophy [14]. Pressure overload and high resistance of the aorta to the left ventricular blood transfusion (post load) in resistance training is the reason (Melo et al. [34]).

In fact, both models of exercise training led to the physiological growth of the heart, yet the type is different. Due to the volume overload, endurance training causes eccentric hypertrophy. In contrast, due to pressure overload, resistance training causes concentric hypertrophy of the heart [37].

In terms of the physiological growth of the heart and the effects of exercise, most research focused solely on cardiomyocytes, while exercise is also effective in stimulating healthy heart growth on other types of heart cells [42, 43]. Among these cells are the lesser known telocytes. However, the evaluation of telocytes by transmission electron microscopy (TEM) has been called the gold standard method and using this method to determine the ultrastructural properties of TCs in rats and not using this method is one of the limitations of the present study. But dual staining is the most common tool for quantitative evaluation of telocytes, which can also distinguish telocytes from interstitial cell types [12, 47]. We also used dual staining of CD34 / PDGFRα, CD34 / PDGFRβ and vimentin to determine changes in the number of telocytes in the heart. The results showed a significant increase in the number of telocytes in the hearts of trained rats.

Studies have shown a decrease in the number of cardiac telocytes in the infarcted zone following myocardial infarction [22, 42, 43, 49, 51] and transplantation of telocytes to the heart with myocardial infarction reduces the size of the infarction and improves myocardial function [3, 22, 42, 43, 50, 51]. Cardiac telocytes also help regenerate the heart’s interstitial network and maintain myocardial function, which can be important in providing the right microenvironment for the migration and development of heart stem cells [7]. Manole and colleagues investigated the involvement of telocytes in neo-angiogenesis after myocardial infarction. Using transmission electron microscopy, immunohistochemistry, and analysis of several progenitor microRNAs, they proved that telocytes are closely related to neo-angiogenesis factors. Cardiac telocytes promote NOS2 and VEGF and contain measurable amounts of angiogenic microRNAs. Therefore, they can involve in the process of myocardial angiogenesis through their paracrine effects [32]. Although the heart is said to be a post-mitotic organ because adult cardiomyocytes are unable to regenerate, but the adult heart is a reservoir of cardiac stem cells located mainly in the epicardial cardiogenic niches. They are responsible for the spontaneous renewal of the heart, albeit limited, after injury [9]. On the other hand, in addition to creating a framework in the myocardial stroma, telocytes are also present in cardiogenic niches, where they are closely related to cardiac stem cells, which strongly indicates their function as nurse and support cells. In fact, telocytes appear to participate in sustaining cross-talk, promoting regenerative mechanisms, and supporting differentiation of local stem cell niches [7], Ravalli et al. [36]), thus helping to repair and regenerate the heart. Vukusic and colleagues [45] conducted a study in which they used BrdU labeling and exercise training to investigate a potential cardiac stem cell niche. They identified a potential stem cell niche in atrioventricular junction of the adult rat heart and suggest that exercise training can affect this microenvironment, potentially leading to changes in stem cell behavior and function.

Studies on telocytes’ response to exercise are very limited. Rawalli examined the presence of telocytes in the tibialis anterior muscle of healthy male rats who had done endurance training for 4 or 16 weeks compared with sedentary rats. They showed that regular exercise had protective effects against muscle atrophy. Also in their study, the amount of telocytes in rats that were inactive for 16 weeks was significantly reduced. On the other hand, 16 weeks of endurance training prevented a decrease in muscle telocytes, and although the rate of increase in muscle telocytes was not statistically significant in rats that exercised for 16 weeks compared to the 4-week training group, it was significantly higher than that of 16-week control group. Therefore, their results confirm the present study on the positive effect of regular exercise on telocytes, while emphasizing the role of telocytes in muscle growth and prevention of muscle atrophy (Ravalli et al. [36]).

In the case of cardiac telocytes, Liao and his colleagues investigated the impact of 2 weeks of moderate-intensity running on cardiac telocytes density in female Sprague Dawley rats following myocardial infarction to understand the mechanism underlying the positive effects of exercise. They demonstrated that early moderate exercise significantly increases the number of cardiac telocytes in the border zone and might have a beneficial effect on the survival of cardiomyocytes in this area, however the early moderate exercise had no such effect in the infarct zone [28]. Xiao and his colleagues also trained C57 mice for 4 weeks. The mice swam 10 min a day. Swimming time was increased by 10 min each day. Following 4 weeks of training, they measured cardiac growth factors and the number of cardiac telocytes. The results revealed that endurance training significantly increase the number of telocytes in exercised heart [46]. In line with their results, our experiment also revealed that 8 weeks of endurance and resistance training, both, led to an increase in the number of cardiac telocytes. Then, by activating the cardiac stem cells, telocytes probably involve in the physiological growth of the heart. It seems that activation of several cytokines, chemokines [1, 7], or even microRNAs [11] by these cells leads to the regulation of differentiation and proliferation of heart stem cells. However, another one of the limitations of the present study is that we do not evaluate the molecular mechanisms of possible involvement of telocytes in cardiac stem cell activation following exercise training and therefore more studies are needed in this field.

Finally, we note that the effect of endurance and resistance training on cardiomyocytes and telocytes count was similar. That is, the response of cardiac telocytes does not depend on the type of exercise activity and the type of growth (either concentric or eccentric).

## Data Availability

The datasets used are available from the corresponding author upon request.

## Abbreviations

ANOVA: Analysis of variance
ANP: Atrial natriuretic peptide
BNP: Brain natriuretic peptide
GAPDH: Glyceraldehyde-3-phosphate dehydrogenase
PI: Propidium iodide
qRT-PCR: Quantitative real-time polymerase chain reaction
TEM: Transmission electron microscopy
VO2max: Maximal oxygen uptake
